# Targeted antenatal anti-D prophylaxis for RhD-negative pregnant women: a systematic review

**DOI:** 10.1186/s12884-020-2742-4

**Published:** 2020-02-07

**Authors:** Britta Runkel, Gregor Bein, Wiebke Sieben, Dorothea Sow, Stephanie Polus, Daniel Fleer

**Affiliations:** 1 Institute for Quality and Efficiency in Health Care (IQWiG), Cologne, Germany; 2 Institute for Clinical Immunology and Transfusion Medicine, Justus-Liebig-University, Giessen, Germany; 3 Institute for Research in Operative Medicine, Witten/Herdecke University, Cologne, Germany

**Keywords:** Genotyping techniques, Rh-Hr blood-group system, Fetus, Benefit assessment, Systematic review

## Abstract

**Background:**

All non-sensitized Rhesus D (RhD)-negative pregnant women in Germany receive antenatal anti-D prophylaxis without knowledge of fetal RhD status. Non-invasive prenatal testing (NIPT) of cell-free fetal DNA in maternal plasma could avoid unnecessary anti-D administration. In this paper, we systematically reviewed the evidence on the benefit of NIPT for fetal RhD status in RhD-negative pregnant women.

**Methods:**

We systematically searched several bibliographic databases, trial registries, and other sources (up to October 2019) for controlled intervention studies investigating NIPT for fetal RhD versus conventional anti-D prophylaxis. The focus was on the impact on fetal and maternal morbidity. We primarily considered direct evidence (from randomized controlled trials) or if unavailable, linked evidence (from diagnostic accuracy studies and from controlled intervention studies investigating the administration or withholding of anti-D prophylaxis). The results of diagnostic accuracy studies were pooled in bivariate meta-analyses.

**Results:**

Neither direct evidence nor sufficient data for linked evidence were identified. Meta-analysis of data from about 60,000 participants showed high sensitivity (99.9%; 95% CI [99.5%; 100%] and specificity (99.2%; 95% CI [98.5%; 99.5%]).

**Conclusions:**

NIPT for fetal RhD status is equivalent to conventional serologic testing using the newborn’s blood. Studies investigating patient-relevant outcomes are still lacking.

## Bulleted statements

- what’s already known about this topic? Non-invasive prenatal testing (NIPT) for fetal RhD from maternal plasma may enable targeted anti-D prophylaxis for RhD-negative women carrying an RhD-positive fetus.
- what does this study add? NIPT of fetal RhD shows high sensitivity and specificity and is equivalent to conventional postnatal testing using a blood sample of the newborn.

## Background

During pregnancy, a Rhesus D (RhD)-negative woman may develop antibodies if her fetus is RhD-positive. These maternal allo-antibodies directed against fetal red cell surface antigens that the mother herself lacks can lead to hemolytic disease of the fetus and newborn (HDFN) [1]. Anti-D immunoglobulin (anti-D) administration was introduced in the early 1970s to reduce the incidence of alloimmunization (sensitization) of pregnant women to the D antigen and subsequently the incidence of HDFN, which has since decreased dramatically [2]. In many countries, the current policy is to administer anti-D to non-sensitized RhD-negative pregnant women in the 28th week of gestation [3]. After birth, the cord blood is phenotyped and postnatal anti-D prophylaxis is offered only if the newborn is RhD-positive.

In a Cochrane review of 6 randomized controlled trials (RCTs), postnatal anti-D prophylaxis was shown to be effective in reducing the incidence of sensitization 6 months after birth and in a subsequent pregnancy [2]; the benefits were seen when anti-D was given within 72 h of birth, with higher doses being more effective than lower ones. However, postnatal prophylaxis does not prevent antenatal sensitization [4]. The current policy of universal antenatal anti-D administration leads to approximately 50,000 RhD-negative pregnant women per year in Germany receiving anti-D prophylaxis even though they are carrying an RhD-negative fetus [5].

Non-invasive prenatal testing (NIPT) for fetal RhD from maternal plasma may enable anti-D prophylaxis to be withheld from RhD-negative women carrying an RhD-negative fetus. As early as 1998, Lo et al. [6] described the presence of fetal DNA in maternal plasma and the possibility of non-invasive determination of the fetal RhD status. These findings enable non-invasive, risk-free antenatal testing, which is mostly performed using the real-time polymerase chain reaction (PCR).

The aim of the current article was to systematically review the evidence on the benefit of NIPT for fetal RhD status in RhD-negative pregnant women and subsequent targeted anti-D prophylaxis. The focus of the assessment was on patient-relevant outcomes.

## Methods

### Protocol and methodological approach

IQWiG’s responsibilities and general methods are described in its methods paper [7]. The methods for the present assessment were defined a priori and published in a German-language protocol on the website of the German Institute for Quality and Efficiency in Health Care (IQWiG) [8]. The full German-language report including the original literature search [9], as well as an English-language extract [10], are also available on the website. The report is currently being used to inform a reimbursement decision on future RhD testing in Germany, thus potentially affecting about 750.000 pregnant women per year.

An update search was conducted for the current article, which was written according to the PRISMA statement [11] (see Additional file 1).

### Eligibility criteria

The target population comprised non-sensitized RhD-negative pregnant women investigated in controlled intervention studies of the diagnostic-therapeutic chain. The test intervention was NIPT for fetal RhD, with subsequent administration or withholding of anti-D prophylaxis, depending on the test result. The control intervention was conventional anti-D prophylaxis for all non-sensitized RhD-negative pregnant women using the anti-D dose approved in Germany. The patient-relevant outcomes investigated included rates of mortality, HDFN and adverse events as well as health-related quality of life (if meaningful, referring to both maternal and fetal or pediatric outcomes). Sensitization rates were investigated as a surrogate outcome for HDFN.

If the kind of direct evidence described above was not available, we planned to apply a linked evidence approach [12].

We considered the following evidence and study types:

Either direct evidence from RCTs of the diagnostic-therapeutic chain (if not available, prospective intervention studies were also considered). Or, if no direct evidence was available, linked evidence [12] from studies on diagnostic accuracy, together with controlled intervention studies investigating the benefit (prevention of sensitization) and harm (adverse events) of antenatal anti-D prophylaxis. The detailed eligibility criteria are presented in Table 1.

**Table 1:** Eligibility criteria

	Direct evidence	Linked evidence		
	intervention studies	diagnostic accuracy study	intervention studies	
Population	• RhD-negative pregnant women without sensitization	• RhD-negative pregnant women without sensitization	• RhD-negative pregnant women without sensitization	
Study intervention	• non-invasive prenatal RhD-testing of the fetus and omission of antenatal anti-D prophylaxis in the case of an RhD- negative fetus	• non-invasive prenatal RhD-testing of the fetus	• administration of anti-D prophylaxis	
Control intervention	• anti-D prophylaxis for all RhD-negative pregnant women	• postnatal RhD-testing of the newborn	• no antenatal administration of anti-D prophylaxis	
			Benefits	Harms
Patient-relevant outcomes/diagnostic accuracy measures	• mortality	• test accuracy (sensitivity, specificity, false-negative rate, false-positive rate)	• mortality	• mortality
	• HDFN (surrogate outcome: sensitization)		• HDFN (surrogate outcome: sensitization)	• adverse events
	• adverse events			• health-related quality of life
	• health-related quality of life		• health-related quality of life	
Study type	• RCTs	• prospective cohort studies	• RCTs	• RCTs
	• prospective, non-randomized controlled intervention studies		• prospective, non-randomized controlled intervention studies	• prospective, non-randomized controlled intervention studies
				• cohort studies (also retrospective or with historical controls)

HDFN: hemolytic disease of the fetus and newborn

### Search strategy and study selection

We searched for relevant primary studies and secondary publications (systematic reviews and HTA reports) in MEDLINE (1946 to October 2019) and EMBASE (1974 to October 2019) via Ovid as well as in the Cochrane Central Register of Controlled Trials (October 2019). The Cochrane Database of Systematic Reviews (Cochrane Reviews), the Database of Abstracts of Reviews of Effects (Other Reviews), and the Health Technology Assessment Database (Technology Assessments) were searched for relevant secondary publications. In addition, we screened web-based trial registries (ClinicalTrials.gov, International Clinical Trials Registry Platform Search Portal, and the EU Clinical Trials Register). The search strategy, which was developed by one information specialist and checked by another, is presented in Additional file 2. We also screened the websites of the European Medicines Agency and the US Food and Drug Administration.

Two reviewers independently screened titles and abstracts of the citations retrieved to identify potentially eligible primary and secondary publications. The full texts of these articles were obtained and independently evaluated by the same two reviewers applying the full set of inclusion and exclusion criteria. Disagreements were resolved by consensus. Study selection was performed in IQWiG’s internal web-based trial selection database (webTSDB) [13]. Endnote X9 was used for citation management.

### Data extraction

The individual steps of the data extraction and risk-of-bias assessment procedures were always conducted by one person and checked by another; disagreements were resolved by consensus. Details of the studies were extracted using standardized tables developed and routinely used by IQWiG. Depending on the study question (comparison of interventions or evaluation of diagnostic accuracy) we extracted information on study design, sample size, patient-relevant outcomes or diagnostic accuracy, location and period during which the study was conducted, dropout rate, gestational age, treatment regimen and control treatment or index test and reference standard, as well as risk-of-bias items (see below).

### Assessment of risk of bias

We assessed the risk of bias for individual studies, as well as for each outcome, and rated these risks as “high” or “low”.

For controlled intervention studies, the risk of bias was assessed by determining the adequacy of the following quality criteria, which closely follow the criteria of the Cochrane risk-of-bias tool [14]): generation of random allocation sequence or whether both treatment groups were studied in parallel, allocation concealment or comparability of groups, blinding of participants and investigators, as well as selective outcome reporting. If the risk of bias on the study level was rated as “high”, the risk of bias on the outcome level was also generally rated as “high”. The risk of bias for each outcome was assessed by determining the adequacy of the following quality criteria: blinding of outcome assessors, application of the intention-to-treat (ITT) principle, and selective outcome reporting.

For studies on diagnostic accuracy, the risk of bias was assessed by determining the adequacy of the following quality criteria following QUADAS-2 [15]: patient selection, index test, reference standard, as well as flow and timing. Concerns about applicability were assessed by determining the adequacy of the following quality criteria: patient selection, index test and reference standard.

The risk of bias determines the confidence in the conclusions drawn from the study data and can be used to explore possible reasons for heterogeneity if the studies differ in their risk of bias.

### Data analysis

For the statistical analysis of controlled intervention studies, we used the results from the ITT analysis. We reported the treatment effects as odds ratios (ORs), including 95% confidence intervals (CIs), for binary outcomes. We conducted a random effects meta-analysis of intervention studies using the Knapp-Hartung method [16] as well as sensitivity analyses using the Mantel-Haenszel method and a Beta-binomial model. No subgroup analyses were conducted.

Separate meta-analyses were performed to pool the results of diagnostic accuracy studies. Sensitivities and specificities were summarized in a bivariate meta-analysis. Model parameters were estimated by means of a generalized linear mixed model. No sensitivity or subgroup analyses were conducted.

All calculations were performed with the statistical software SAS.

## Results

### Literature search (see Figs. 1 and 2 for flowchart)

Overall, 2237 studies were screened. No studies of the diagnostic-therapeutic chain were identified. 70 studies on diagnostic accuracy including approximately 66,000 participants were identified (all in bibliographic databases), of which the 12 largest (including over 90% of the total study population) were included in the analysis [5, 17–28]. Two controlled intervention studies investigating the benefit (prevention of sensitization) of antenatal anti-D prophylaxis were identified (in bibliographic databases). However, they used a low and non-approved dose for anti-D prophylaxis [29, 30]. The results of these off-label studies are described below. No studies investigating harm (adverse events) from anti-D prophylaxis were identified.

**Fig. 1:**
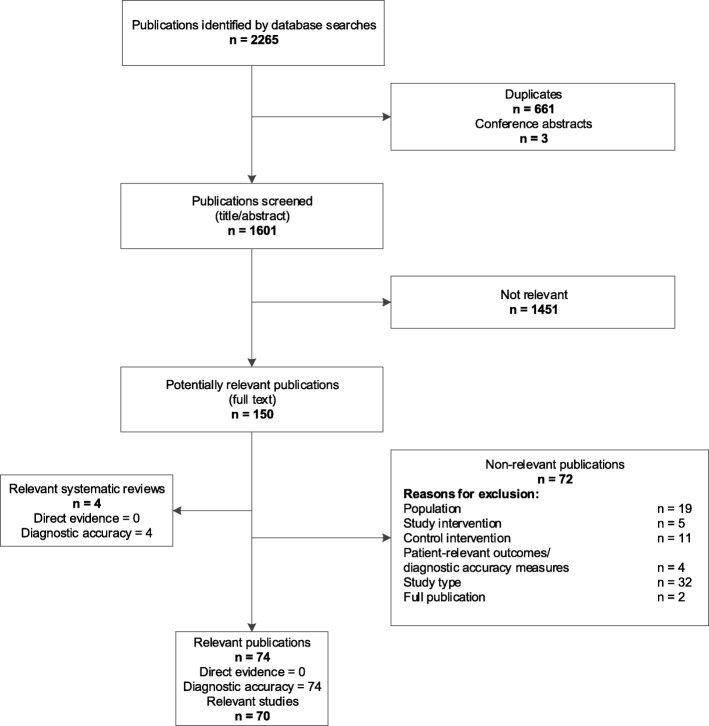
Flowchart of study selection for direct trial evidence and linked evidence (diagnostic accuracy studies)

**Fig. 2:**
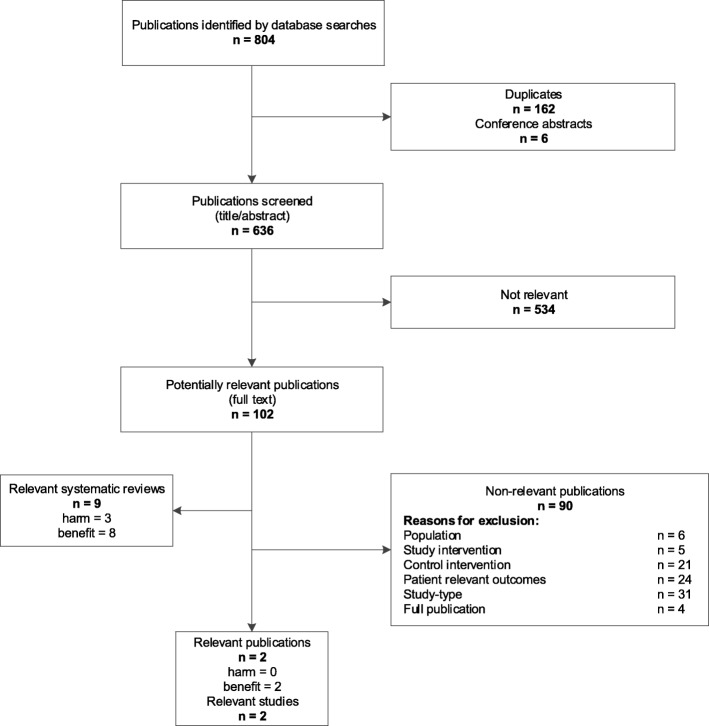
Flowchart of study selection for linked evidence (controlled intervention studies – benefit and harm of anti-D prophylaxis)

### Study characteristics

Table 2 presents the main characteristics of the 12 largest diagnostic accuracy studies and the two off-label studies on anti-D prophylaxis.

**Table 2:** Study characteristics

Study	Study design	Participants (intervention/control)	Treatment/index test	Patient-relevant outcomes/ reference test	Location/recruitment period	Weeks’ gestation Median [min; max]	Drop-out (intervention/control)
Huchet 1987 [29]	prospective intervention study	1969 (927/955) with RhD-positive newborns: (599/590)	100 μg anti-D immunoglobulin, one dose at 26 to 29 and one at 32–36 weeks’ gestation	sensitization	23 hospitals in the Paris region 01/1983–06/1984		Not stated
Lee 1995 [30]	RCT	2541 (1268/1273) with RhD-positive newborns: (513/595)	250 IU anti-D immunoglobulin at 28 and 34 weeks’ gestation	sensitization	Multi-center study in UK Not stated		642 (362/280)
De Haas 2016 [17]	prospective cohort study	32,222	cff-DNA *RHD* Exons 5 and 7	serologic cord blood testing	Netherlands (national screening program) 07/2011–10/2012	Mean in weeks + days [SD] 27 + 6 [0 + 6] [min; max] [27; 29]	6433
Clausen 2014 [18]	prospective cohort study	14,547	cff-DNA *RHD* Exons 5, 7 or 10	serologic cord blood testing	Denmark (national screening program) 01/2010 for 2 years	25 [n. a.]	1879
Haimila 2017 [19]	prospective cohort study	10,814	cff-DNA *RHD* Exons 5 and 7	serologic cord blood testing / heel stick	Finland (national screening program) 02/2014–01/2016	n. a. [24; 26]	0
Wikman 2012 [20]	prospective cohort study	4118	cff-DNA *RHD* Exon 4	serologic cord blood testing / blood sample of newborn	Sweden 09/2009–05/2011	10 [3; 40]	466
Chitty 2014 [21]	prospective cohort study	3039	cff-DNA *RHD* Exons 5 and 7	serologic cord blood testing	England 2009–2012	19 [5; 35]	781
Finning 2008 [22]	prospective cohort study	1997	cff-DNA *RHD* Exons 5 and 7	serologic cord blood testing	England/not stated	28 [8; 38]	128
Müller 2008 [5]	prospective cohort study	1113	cff-DNA *RHD* Exons 5 and 7	serologic cord blood testing	Germany 2006 – not stated	25 [6; 32]	91
Macher 2012 [23]	prospective cohort study	1012	cff-DNA *RHD* Exons 5 and 7	serologic cord blood testing	Spain 2010	n.a. [10; 28]	0
Hyland 2017 [24]	prospective cohort study	665	cff-DNA RHD Exon 5 and 10	serologic cord blood testing	Australia Not stated	19.3 [9; 37]	66
Akolekar 2011 [26]	prospective cohort study	591	cff-DNA *RHD* Exons 5 and 7	serologic cord blood testing	UK Not stated	12,4 [11; 14]	5
Minon 2008 [27]	prospective cohort study	563	cff-DNA *RHD* Exons 4, 5 and 10	serologic cord blood testing	Belgium 11/2002–12/2006	17,5 [10; 38]	Not stated
Soothill 2015 [28]	prospective cohort study	529	cff-DNA *RHD* Exons 5 and 7	serologic cord blood testing	England 04–09/2013	Not stated	30

*cff* cell-free fetal, *n.a* not available, *RHD* rhesus factor, *SD* standard deviation

### Risk of bias

Both off-label studies on anti-D prophylaxis showed a high risk of bias on the study and outcome level, for example, because of unclear information on the blinding of patients and investigators and/or an inappropriate ITT analysis. In 11 of the 12 diagnostic accuracy studies, the risk of bias was high in the total score (Table 3). However, the pooled estimate of all studies were similar to the results of the study with the low risk of bias.

**Table 3:** Risk of bias of included studies (QUADAS 2) and concerns regarding applicability

Study	Patient selection	Index test	Reference standard	Flow and timing	Applicability concerns - total
De Haas 2016	low	unclear	low	high	low
Clausen 2014	low	unclear	unclear	high	low
Haimila 2017	low	unclear	unclear	low	low
Wikman 2012	low	unclear	unclear	high	low
Chitty 2014	unclear	low	unclear	high	low
Finning 2008	unclear	unclear	low	low	low
Müller 2008	low	unclear	unclear	low	low
Macher 2012	low	unclear	unclear	low	low
Hyland 2017	low	unclear	unclear	low	low
Akolekar 2011	unclear	unclear	unclear	low	low
Minon 2008	low	unclear	unclear	low	low
Soothill 2015	low	low	low	low	low

### Effects of antenatal anti-D prophylaxis

The meta-analysis of the results of the two off-label studies (Additional file 3) showed no significant differences in sensitization at the time of delivery (OR 0.33, 95% CI [0; 123,851], number of participants = 2297, number of studies = 2, I^2^ = 51%). The CI is very wide and the effect could not be estimated with adequate precision. We therefore conducted different sensitivity analyses with 2 different meta-analysis methods, the Mantel-Haenszel (MH) method and the beta-binomial model (BBM). Both led to more precise estimates (MH: 0.37 [0.13; 1.06], number of participants = 2297, number of studies = 2, I^2^ = 51%; BBM 0.30 [0.07; 1.26], number of participants = 2297, number of studies = 2), but neither showed a significant difference between the test and control groups.

### Diagnostic accuracy

Sensitivities and specificities from the 12 studies are described comparatively in Table 4. The bivariate meta-analysis showed high values for both measures of diagnostic accuracy of NIPT in RhD-negative pregnant women (sensitivity: 99.9% (95% CI [99.5%; 100%]; specificity: 99.2% (95% CI [98.5%; 99.5%], number of participants = 60,011, number of studies = 12). Two of the studies [5, 17] assessed discordant results of ante- and postnatal tests by genetic testing. They found that the postnatal test also produced a few incorrect test results (about 35 false-negative results out of 27,000 tests due to RhD variants or confusion of the samples), indicating that both tests can be regarded as equivalent.

**Table 4:** Diagnostic accuracy results

Study	n	TP	FN	FP	TN	Inconclusive results (%)^a, b^	Sensitivity in % [95% CI]^b^	Specificity in % [95% CI]^b^
De Haas 2016	25,789	15,816	9	225	9739	0 (0)^c^	99.9 [99.9; 100]	97.7 [97.4; 98.0]
Clausen 2014	12,668	7636	11	41	4706	274 (2.2)	99.9 [99.7; 99.9]	99.1 [98.8; 99.4]
Haimila 2017	10,814	7080	1	7	3640	86 (0.80)	100 [99.9; 100]	99.8 [99.6; 99.9]
Wikman 2012	3652	2236	55	15	1331	15^b^ (0.4)	97.6 [96.9; 98.2]	98.9 [98.2; 99.4]
Chitty 2014	956^d^	535	1	4	341	75 (7.8)	99.8 [99.0; 100]	98.8 [97.1; 99.7]
	2288^e^	2563	19	18	1920	393 (17.2)	99.3 [98.9; 99.6]	99.1 [98.5; 99.4]
Finning 2008	1869	1118	3	14	670	64 (3.4)	99.7 [99.2; 99.9]	98.0 [96.6; 98.9]
Müller 2008	1022							
“Spin column”^f^		660^b^	2^b^	3^b^	357^b^	0 (0)^b^	99.7 [98.9; 100]	99.2 [97.6; 99.8]
“Magnetic tips”^f^		661^b^	1^b^	7^b^	353^b^	0 (0)^b^	99.8 [99.2; 100]	98.1 [96.0; 99.2]
Macher 2012	1012	619	0	7	386	0 (0)	100 [99.4; 100]	98.2 [96.4; 99.3]
Hyland 2017	599	370	0	1	226	2 (0.3)^b^	100 [99.0; 100]	99.6 [97.6; 100]
Akolekar 2011	586	332	6	0	164	84 (14.3)	98.2 [96.2; 99.3]	100 [97.8; 100]
Minon 2008	545	360	0	0	185	0 (0)	100 [99.0; 100]	100 [98.0; 100]
Soothill 2015	499	267	0	1	170	61^g^ (12.2)	100 [98.6; 100]	99.4 [96.8; 100]
						pooled estimate^h^	99.9 [99.5; 100]	99.2 [98.5; 99.5]

a: Proportion of study participants with inconclusive results

b: IQWiG’s own calculation

c: 0.21% of samples were inconclusive (women with RhD variants). In this study these samples were categorized by the positive samples

d: Results of the largest cohort of this study (11 to 13 weeks’ gestation). These results are included in the pooled effect

e: Summarized data for 2288 evaluated women with a total of 4913 data sets including up to 4 measurement points (multiple measurements). The number of blood samples is therefore shown here

f: “Spin column” and “magnetic tips” are two different methods for the extraction of cff-DNA from plasma samples. The patients with samples extracted by the spin column method are included in the pooled effect

g: Treated like positive samples

h: Generalized linear model to take into account the dependency between sensitivity and specificity

cff: cell-free fetal; FN: false negative; FP: false positive; CI: confidence interval; n: number of evaluated participants; RHD: rhesus factor; TN: true negative; TP: true positive

## Discussion

The current review shows a lack of studies investigating patient-relevant outcomes after NIPT for fetal RhD status in RhD-negative pregnant women and subsequent targeted anti-D prophylaxis. The analysis of diagnostic accuracy studies shows that NIPT has a high sensitivity and specificity.

### Comparison with the literature

#### Anti-D prophylaxis

The Cochrane review by McBain 2015 [4] included the same two off-label studies on antenatal anti-D prophylaxis described in our review [29, 30]. In accordance with our findings, the authors stated that these two studies do not provide conclusive evidence that the use of anti-D during pregnancy shows a benefit in terms of incidence of Rhesus D sensitization.

A systematic review by Pilgrim 2009 [31] contained 12 studies (including one of the off-label studies [29] described in our review) with a high risk of bias, such as studies with historical controls, retrospective studies, and community intervention trials. They concluded that antenatal anti-D prophylaxis may reduce the incidence of sensitization. Furthermore, they noted that anti-D is associated with only minimal adverse effects.

In a systematic review by Turner 2012 [32], a pooled OR of 0.31 (95% CI [0.17; 0.56]) was determined in an adjusted meta-analysis of 10 studies on the administration of antenatal anti-D prophylaxis and the incidence of sensitization. Among these were the two off-label studies described in our review and further studies with historical control groups. The authors concluded that there was strong evidence of the effectiveness of routine antenatal anti-D prophylaxis for prevention of sensitization.

#### Diagnostic accuracy

We identified 70 relevant studies on diagnostic accuracy, of which 58 included only a comparatively small number of participants (2 to 467). We therefore restricted our sample to the 12 largest studies, which comprised over 90% of the overall study population. A sufficiently accurate determination of the diagnostic accuracy of NIPT for fetal RhD was thus possible, showing high sensitivity and specificity.

Mackie 2017 [33] included 30 studies and found a sensitivity of 99.3% (95% CI [98.2, 99.7%]) and a specificity of 98.4% (95% CI [96.4, 99.3%]). These results are comparable to our findings, despite a differing study pool (only 2 of the 30 studies were included in our review).

A British National Institute for Health and Care Excellence (NICE) report from 2016 [34, 35] on diagnostic accuracy included eight studies exclusively using “high throughput” NIPT (six of these studies were included in our review). The corresponding HTA report [36] found that after 11 weeks of pregnancy only 1% of the samples showed an incorrect test result (almost all false-positive) and approximately 7% of the samples showed an inconclusive result. A pooled rate of false-negative results of 0.34% [95% CI [0.15%; 0.76%)] was reported, which is comparable to the sensitivity determined in our review (99.9% [95% CI [99.5%; 100%]). According to NICE, if antenatal anti-D prophylaxis was administered only to RhD-negative pregnant women with RhD-positive fetuses, this would result in potential cost savings between £296,000 and £409,000 per 100,000 pregnancies [36, 37]. NICE has issued a positive recommendation for NIPT [38].

A French Haute Autorité de Santé (HAS) report on diagnostic accuracy from 2011 [39, 40] is based on 31 studies, which were not pooled in a meta-analysis. Despite the differing study pools (only two studies were included in our review), their results are comparable: the majority of the studies included (22 of 31) reported a sensitivity and specificity of over 95%. HAS concluded that the expected benefit of NIPT was sufficient to justify reimbursement by the health insurance funds, and it is now being reimbursed in France. They recommend applying the test between the 11th and 28th week of pregnancy.

### Limitations

The meta-analysis of diagnostic accuracy was limited by fact that the true fetal RhD status could not be determined by genetic testing in the primary studies. Only two studies resolved discrepancies between the ante- and postnatal test. As postnatal testing can also be incorrect, using postnatal test results as the reference standard might underestimate the true accuracy of the prenatal test. An additional limitation of the present review was the restriction of analyses to only the largest primary studies. However, the inclusion of all studies, regardless of sample size, would probably not have altered the main findings. Furthermore, the non-publication of negative findings is more common in smaller studies [41], so focusing on larger studies reduces bias.

### Ethical aspects

With the implementation of NIPT for fetal RhD status, almost 40% of antenatal anti-D administrations could be saved per year in Germany [5]. Important aspects are not only the costs, but also ethical issues concerning the acquisition of anti-D: male donors are sensitized with a blood product to produce the vaccine and the number of donors worldwide is limited; most countries rely on imports.

## Conclusion

In summary, NIPT for fetal RhD status shows high sensitivity and specificity and is equivalent to conventional postnatal testing using a blood sample of the newborn, which also produces a few incorrect test results. Some countries (e.g. Denmark and Netherlands) have already implemented NIPT and have abolished postnatal testing. However, as studies investigating the effects of NIPT on patient-relevant outcomes are still lacking, before its widespread implementation as the only test to determine RhD status, we recommend evaluating the benefit of NIPT in the respective health care settings.

## Supplementary information

**Additional file 1.** PRISMA checklist

**Additional file 2.** Search strategy

**Additional file 3: Table S5**. Effects of antenatal anti-D prophylaxis

## Data Availability

All data generated or analysed in this research are included in this published article or in the full German-language report, https://www.iqwig.de/download/D16-01_Bestimmung-fetaler-Rhesusfaktor_Abschlussbericht_V1-0.pdf

## Abbreviations

BBM: beta-binomial model
CI: Confidence interval
DNA: Deoxyribonucleic acid
HAS: Haute Autorité de Santé
HDFN: Hemolytic disease of the fetus and newborn
HTA: Health Technology Assessment
IQWiG: Institute for Quality and Efficiency in Health Care
ITT: Intention-to-treat
MH: Mantel-Haenszel
NICE: National Institute for Health and Care Excellence
NIPT: Non-invasive prenatal testing
OR: Odds ratio
PCR: Polymerase chain reaction
PRISMA: Preferred Reporting Items for Systematic Reviews and Meta-Analyses
QUADAS-2: Quality Assessment of Diagnostic Accuracy Studies 2
RCT: Randomized controlled trial
RhD: Rhesus D
webTSDB: Web-based trial selection database
