# Supplementary material for: Targeted antenatal anti-D prophylaxis for RhD-negative pregnant women: a systematic review
Source: BMC Pregnancy Childbirth. 2020 Feb 7;20:83. doi: 10.1186/s12884-020-2742-4 (PMC7006196; doi:10.1186/s12884-020-2742-4)
Supplement: Supplementary file 2 — Additional file 2. Search strategy [file 12884_2020_2742_MOESM2_ESM.docx]

**Additional file 2:**

**Search strategy for direct evidence and linked evidence (diagnostic accuracy)**

**1. EMBASE**

***Search interface: Ovid***

- Embase 1974 to 2019 October 10

| # | Searches |
| --- | --- |
| 1 | Fetus/ |
| 2 | fetus blood sampling/ |
| 3 | (fetal* or fetus* or foetal*).ti,ab. |
| 4 | or/1-3 |
| 5 | exp rhesus antibody/ |
| 6 | rhesus D antigen/ |
| 7 | blood group rhesus system/ |
| 8 | rhesus incompatibility/ |
| 9 | (RHD* or "rhesus D").ti,ab. |
| 10 | or/5-9 |
| 11 | ec.fs. |
| 12 | maternal plasma/ |
| 13 | maternal blood/ |
| 14 | genotyping*.ti,ab. |
| 15 | (maternal adj3 (plasma* or blood* or serum*)).ti,ab. |
| 16 | (cffDNA* or DNA*).ti,ab. |
| 17 | or/11-16 |
| 18 | and/4,10,17 |
| 19 | 18 not (exp animal/ not exp humans/) |
| 20 | 19 not (Conference Abstract or Conference Review or Editorial).pt. |

**2. MEDLINE**

***Search interface: Ovid***

- Ovid MEDLINE(R) 1946 to October Week 1 2019
- Ovid MEDLINE(R) Daily Update October 10, 2019
- Ovid MEDLINE(R) In-Process & Other Non-Indexed Citations 1946 to October 10, 2019
- Ovid MEDLINE(R) Epub Ahead of Print October 10, 2019

| # | Searches |
| --- | --- |
| 1 | exp Fetus/ |
| 2 | Fetal Diseases/ |
| 3 | (fetal* or fetus* or foetal*).ti,ab. |
| 4 | or/1-3 |
| 5 | Rh-Hr Blood-Group System/ |
| 6 | Rh Isoimmunization/ |
| 7 | (RHD* or "rhesus D").ti,ab. |
| 8 | or/5-7 |
| 9 | blood.fs. |
| 10 | genetics.fs. |
| 11 | genotyping*.ti,ab. |
| 12 | (maternal adj3 (plasma* or blood* or serum*)).ti,ab. |
| 13 | (cffDNA* or DNA*).ti,ab. |
| 14 | or/9-13 |
| 15 | and/4,8,14 |
| 16 | 15 not (comment or editorial).pt. |

**3. PubMed**

***Search interface: NLM***

- PubMed - as supplied by publisher
- PubMed - in process
- PubMed – pubmednotmedline

| Search | Query |
| --- | --- |
| #1 | Search fetal* [TIAB] OR fetus* [TIAB] OR foetal* [TIAB] |
| #2 | Search RHD* [TIAB] OR "rhesus D"[TIAB] |
| #3 | Search genotyping*[TIAB] |
| #4 | Search maternal [TIAB] AND (plasma* [TIAB] OR blood* [TIAB] OR serum* [TIAB]) |
| #5 | Search cffDNA*[TIAB] OR DNA*[TIAB] |
| #6 | Search #3 OR #4 OR #5 |
| #7 | Search #1 AND #2 AND #6 |
| #8 | Search #7 NOT Medline[sb] |

**4. The Cochrane Library**

***Search interface for the first search: Wiley***

- Cochrane Database of Systematic Reviews: Issue 1 of 12, January 2017
- Database of Abstracts of Reviews of Effect: Issue 2 of 4, April 2015
- Cochrane Central Register of Controlled Trials: Issue 11 of 12, November 2016
- Health Technology Assessment Database: Issue 4 of 4, October 2016

| ID | Search |
| --- | --- |
| #1 | [mh Fetus] |
| #2 | [mh ^"Fetal Diseases"] |
| #3 | (fetal* or fetus* or foetal*):ti,ab |
| #4 | fetal* or fetus* or foetal* |
| #5 | #1 or #2 or #3 |
| #6 | #1 or #2 or #4 |
| #7 | [mh ^"Rh-Hr Blood-Group System"] |
| #8 | [mh ^"Rh Isoimmunization"] |
| #9 | (RHD* or "rhesus D"):ti,ab |
| #10 | RHD* or "rhesus D" |
| #11 | #7 or #8 or #9 |
| #12 | #7 or #8 or #10 |
| #13 | Any MeSH descriptor with qualifier(s): [Blood - BL] |
| #14 | Any MeSH descriptor with qualifier(s): [Genetics - GE] |
| #15 | genotyping*:ti,ab |
| #16 | (maternal near/3 (plasma* or blood* or serum*)):ti,ab |
| #17 | cffDNA* or DNA*:ti,ab |
| #18 | genotyping* |
| #19 | maternal near/3 (plasma* or blood* or serum*) |
| #20 | cffDNA* or DNA* |
| #21 | #13 or #14 or #15 or #16 or #17 |
| #22 | #13 or #14 or #18 or #19 or #20 |
| #23 | #5 and #11 and #21 in Cochrane Reviews (Reviews and Protocols) |
| #24 | #5 and #11 and #21 in Trials |
| #25 | #6 and #12 and #22 in Other Reviews |
| #26 | #6 and #12 and #22 in Technology Assessments |

***Search interface for the update searches: Wiley***

- Cochrane Database of Systematic Reviews : Issue 10 of 12, October 2019
- Cochrane Central Register of Controlled Trials : Issue 10 of 12, October 2019

| ID | Search |
| --- | --- |
| #1 | [mh Fetus] |
| #2 | [mh ^"Fetal Diseases"] |
| #3 | (fetal* or fetus* or foetal*):ti,ab |
| #4 | #1 or #2 or #3 |
| #5 | [mh ^"Rh-Hr Blood-Group System"] |
| #6 | [mh ^"Rh Isoimmunization"] |
| #7 | (RHD* or "rhesus D"):ti,ab |
| #8 | #5 or #6 or #7 |
| #9 | Any MeSH descriptor with qualifier(s): [Blood - BL] |
| #10 | Any MeSH descriptor with qualifier(s): [Genetics - GE] |
| #11 | genotyping*:ti,ab |
| #12 | (maternal near/3 (plasma* or blood* or serum*)):ti,ab |
| #13 | cffDNA* or DNA*:ti,ab |
| #14 | #9 or #10 or #11 or #12 or #13 |
| #15 | #4 and #8 and #14 in Cochrane Reviews (Reviews and Protocols) |
| #16 | #4 and #8 and #14 in Trials |

5. Health Technology Assessment Database

Search interface for the update searches: Centre for Reviews and Dissemination

- HTA

| Line | Search |
| --- | --- |
| 1 | (MeSH DESCRIPTOR Fetus) |
| 2 | (MeSH DESCRIPTOR Fetal Diseases) |
| 3 | (fetal* or fetus* or foetal*) |
| 4 | (#1 OR #2 OR #3) |
| 5 | (RHD* or rhesus D or RH*) |
| 6 | #4 AND #5 |
| 7 | (#6) IN HTA |

**Search strategy for linked evidence (controlled intervention studies)**

**1. EMBASE**

***Search interface: Ovid***

- Embase 1974 to 2019 October 10

The following search filters were applied:

- Systematic review: Wong [1] – High sensitivity strategy
- RCT: Wong [1] – Strategy minimizing difference between sensitivity and Specificity
- Adverse Events: Golder [3] – Most sensitive search strategies excluding use of specified adverse effects

| # | Searches |
| --- | --- |
| 1 | blood group rhesus system/ |
| 2 | rhesus isoimmunization/ |
| 3 | rhesus incompatibility/ |
| 4 | rhesus immunization/ |
| 5 | (RHD* or "rhesus D").ti,ab. |
| 6 | or/1-5 |
| 7 | immunoglobulin/ |
| 8 | exp rhesus antibody/ |
| 9 | ((anti-d or RH) adj3 (immun* or gamma*)).ti,ab. |
| 10 | ((prophyla* or prevention*) adj3 (RH* or rhesus* or anti-d)).ti,ab. |
| 11 | (rh adj3 (immunisa* or immuniz* or immunoprophylax*)).ti,ab. |
| 12 | or/7-11 |
| 13 | immunoglobulin/ae, to [Adverse Drug Reaction, Drug Toxicity] |
| 14 | exp rhesus antibody/ae, to |
| 15 | immunoglobulin D antibody/ae, to |
| 16 | or/13-15 |
| 17 | or/9-11 |
| 18 | (safe or safety or side-effect* or undesirable effect* or treatment emergent or tolerability or toxicity or adrs or (adverse adj2 (effect or effects or reaction or reactions or event or events or outcome or outcomes))).ti,ab. |
| 19 | 6 and (16 or (17 and 18)) |
| 20 | (random* or double-blind*).tw. |
| 21 | placebo*.mp. |
| 22 | or/20-21 |
| 23 | (meta analysis or systematic review or MEDLINE).tw. |
| 24 | 6 and 12 and (22 or 23) |
| 25 | or/19,24 |
| 26 | 25 not medline.cr. |
| 27 | 26 not (exp animal/ not exp humans/) |
| 28 | 27 not (Conference Abstract or Conference Review or Editorial).pt. |

**2. MEDLINE**

***Search interface: Ovid***

- Ovid MEDLINE(R) 1946 to October Week 1 2019
- Ovid MEDLINE(R) Daily Update October 10, 2019
- Ovid MEDLINE(R) In-Process & Other Non-Indexed Citations 1946 to October 10, 2019
- Ovid MEDLINE(R) Epub Ahead of Print October 10, 2019

The following search filters were applied:

- Systematic review: Wong [1] – High specifity strategy
- RCT: Lefebvre [2] – Cochrane Highly Sensitive Search Strategy for identifying randomized trials in MEDLINE: sensitivity-maximizing version (2008 revision)
- Adverse Events: Golder [3] – Most sensitive search strategies excluding use of specified adverse effects

| # | Searches |
| --- | --- |
| 1 | Rh-Hr Blood-Group System/ |
| 2 | Rh Isoimmunization/ |
| 3 | (RHD* or "rhesus D").ti,ab. |
| 4 | or/1-3 |
| 5 | gamma-Globulins/ |
| 6 | "Rho(D) Immune Globulin"/ |
| 7 | Immunoglobulin G/ |
| 8 | or/5-7 |
| 9 | (prevention & control or "therapeutic use").fs. |
| 10 | and/8-9 |
| 11 | ((anti-d or RH) adj3 (immun* or gamma*)).ti,ab. |
| 12 | ((prophyla* or prevention*) adj3 (RH* or rhesus* or anti-d)).ti,ab. |
| 13 | (rh adj3 (immunisa* or immuniz* or immunoprophylax*)).ti,ab. |
| 14 | or/10-13 |
| 15 | (ae or co or de).fs. |
| 16 | (safe or safety or side-effect* or undesirable effect* or treatment emergent or tolerability or toxicity or adrs or (adverse adj2 (effect or effects or reaction or reactions or event or events or outcome or outcomes))).ti,ab. |
| 17 | or/15-16 |
| 18 | Randomized Controlled Trial.pt. |
| 19 | Controlled Clinical Trial.pt. |
| 20 | (randomized or placebo or randomly or trial or groups).ab. |
| 21 | drug therapy.fs. |
| 22 | or/18-21 |
| 23 | exp animals/ not humans/ |
| 24 | 22 not 23 |
| 25 | cochrane database of systematic reviews.jn. |
| 26 | (search or MEDLINE or systematic review).tw. |
| 27 | meta analysis.pt. |
| 28 | or/25-27 |
| 29 | 4 and 14 and (17 or 24 or 28) |
| 30 | 29 not (comment or editorial).pt. |

**3. PubMed**

***Search interface: NLM***

- PubMed - as supplied by publisher
- PubMed - in process
- PubMed – pubmednotmedline

| Search | Query |
| --- | --- |
| #1 | Search RHD* [TIAB] OR "rhesus D"[TIAB] |
| #2 | Search (anti-d[tiab] or RH[tiab]) AND (immun*[tiab] or gamma*[tiab]) |
| #3 | Search (prophyla*[TIAB] or prevention*[TIAB]) AND (RH*[TIAB] or rhesus*[TIAB] or anti-d[TIAB]) |
| #4 | Search rh[TIAB] AND (immunisa*[TIAB] or immuniz*[TIAB] or immunoprophylax*[TIAB]) |
| #5 | Search #2 OR #3 OR #4 |
| #6 | Search (safe [TIAB] OR safety [TIAB] OR side-effect* [TIAB] OR undesirable effect* [TIAB] OR treatment emergent [TIAB] OR tolerability [TIAB] OR toxicity [TIAB] OR adrs [TIAB] OR (adverse [TIAB] AND (effect [TIAB] OR effects [TIAB] OR reaction [TIAB] OR reactions [TIAB] OR event [TIAB] OR events [TIAB] OR outcome [TIAB] OR outcomes [TIAB]))) |
| #7 | Search clinical trial*[tiab] or random*[tiab] or placebo[tiab] or trial[ti] |
| #8 | Search search[tiab] or meta analysis[tiab] or MEDLINE[tiab] or systematic review[tiab] |
| #9 | Search #1 AND #5 AND (#6 OR #7 OR #8) |
| #10 | Search #9 NOT Medline[sb] |

**4. The Cochrane Library**

***Search interface for the first search: Wiley***

- Cochrane Database of Systematic Reviews: Issue 2 of 12, February 2017
- Database of Abstracts of Reviews of Effect: Issue 2 of 4, April 2015
- Cochrane Central Register of Controlled Trials: Issue 1 of 12, January 2017
- Health Technology Assessment Database: Issue 4 of 4, October 2016

| ID | Search |
| --- | --- |
| #1 | [mh ^"Rh-Hr Blood-Group System"] |
| #2 | [mh ^"Rh Isoimmunization"] |
| #3 | (RHD* or "rhesus D"):ti,ab |
| #4 | RHD* or "rhesus D" |
| #5 | #1 or #2 or #3 |
| #6 | #1 or #2 or #4 |
| #7 | MeSH descriptor: [gamma-Globulins] this term only |
| #8 | MeSH descriptor: [Rho(D) Immune Globulin] this term only |
| #9 | MeSH descriptor: [Immunoglobulin G] this term only |
| #10 | ((anti-d or RH) near/3 (immun* or gamma*)):ti,ab |
| #11 | ((prophyla* or prevention*) near/3 (RH* or rhesus* or anti-d)):ti,ab |
| #12 | (rh near/3 (immunisa* or immuniz* or immunoprophylax*)):ti,ab |
| #13 | #7 or #8 or #9 or #10 or #11 or #12 |
| #14 | (anti-d or RH) near/3 (immun* or gamma*) |
| #15 | (prophyla* or prevention*) near/3 (RH* or rhesus* or anti-d) |
| #16 | rh near/3 (immunisa* or immuniz* or immunoprophylax*) |
| #17 | #7 or #8 or #9 or #14 or #15 or #16 |
| #18 | #5 and #13 in Cochrane Reviews (Reviews and Protocols) |
| #19 | #5 and #13 in Trials |
| #20 | #6 and #17 in Other Reviews |
| #21 | #6 and #17 in Technology Assessments |

***Search interface for the update searches: Wiley***

- Cochrane Database of Systematic Reviews : Issue 10 of 12, October 2019
- Cochrane Central Register of Controlled Trials : Issue 10 of 12, October 2019

| ID | Search |
| --- | --- |
| #1 | [mh ^"Rh-Hr Blood-Group System"] |
| #2 | [mh ^"Rh Isoimmunization"] |
| #3 | (RHD* or "rhesus D"):ti,ab |
| #4 | #1 or #2 or #3 |
| #5 | MeSH descriptor: [gamma-Globulins] this term only |
| #6 | MeSH descriptor: [Rho(D) Immune Globulin] this term only |
| #7 | MeSH descriptor: [Immunoglobulin G] this term only |
| #8 | ((anti-d or RH) near/3 (immun* or gamma*)):ti,ab |
| #9 | ((prophyla* or prevention*) near/3 (RH* or rhesus* or anti-d)):ti,ab |
| #10 | (rh near/3 (immunisa* or immuniz* or immunoprophylax*)):ti,ab |
| #11 | #5 or #6 or #7 or #8 or #9 or #10 |
| #12 | #4 and #11 in Cochrane Reviews (Reviews and Protocols) |
| #13 | #4 and #11 in Trials |

5. Health Technology Assessment Database

Search interface for the update searches: Centre for Reviews and Dissemination

- HTA

| Line | Search |
| --- | --- |
| 1 | (MeSH DESCRIPTOR Rh-Hr Blood-Group System) |
| 2 | (MeSH DESCRIPTOR Rh Isoimmunization) |
| 3 | (RHD* or "rhesus D") |
| 4 | #1 OR #2 OR #3 |
| 5 | (#4) IN HTA |

**Search strategy in study registries**

1. ClinicalTrials.gov

Provider: U.S. National Institutes of Health

- URL: <http://www.clinicaltrials.gov>
- Search interface: Basic Search

| Search strategy |
| --- |
| RHD OR rhesus |

2. EU Clinical Trials Register

Provider: European Medicines Agency

- URL: <https://www.clinicaltrialsregister.eu>/
- Search interface: Basic Search

| Search strategy |
| --- |
| RHD* OR rhesus |

3. International Clinical Trials Registry Platform Search Portal

Provider: World Health Organization

- URL: <http://apps.who.int/trialsearch/>
- Search interface: standard search

| Search strategy |
| --- |
| RHD OR rhesus |

**References**

1. Wong SSL, Wilczynski NL, Haynes RB. Comparison of top-performing search strategies for detecting clinically sound treatment studies and systematic reviews in MEDLINE and EMBASE. J Med Libr Assoc. 2006;94(4):451-5.

2. Lefebvre C, Manheimer E, Glanville J. Searching for studies. In: Higgins JPT, Green S, editors. Cochrane handbook for systematic reviews of interventions. New York: Wiley; 2008. p. 95-150.

3. Golder S, McIntosh HM, Duffy S, Glanville J. Developing efficient search strategies to identify reports of adverse effects in MEDLINE and EMBASE. Health Info Libr J 2006; 23(1): 3-12.
