# Supplementary material for: Targeted antenatal anti-D prophylaxis for RhD-negative pregnant women: a systematic review
Source: BMC Pregnancy Childbirth. 2020 Feb 7;20:83. doi: 10.1186/s12884-020-2742-4 (PMC7006196; doi:10.1186/s12884-020-2742-4)
Supplement: Supplementary file 3 — Additional file 3: Table S5. Effects of antenatal anti-D prophylaxis [file 12884_2020_2742_MOESM3_ESM.docx]

**Additional file 3:**

Table 5: Effects of antenatal anti-D prophylaxis

| Study | Anti-D prophylaxis | |  | No anti-D prophylaxis | |  | Intervention versus control |
| --- | --- | --- | --- | --- | --- | --- | --- |
|  | n | Number of women with sensitization  n (%) |  | n | Number of women with sensitization  n (%) |  | OR [95% CI]  p-value |
| Huchet 1987 | 599 | 0 (0)^a^ |  | 590 | 6 (1,0)^a^ |  | n.a. |
| Lee 1995 | 513 | 4 (0,8)^a^ |  | 595 | 7 (1,2)^a^ |  | n.a. |
|  |  |  |  |  |  |  |  |
|  |  | **Meta-analyses** |  |  |  |  | **Pooled estimates** |
|  |  |  |  |  |  |  | **OR [95% CI]**  **p-value** |
| Primary analysis (Knapp-Hartung method)^a^ | | | | | |  | 0.33 [0;123851]; 0.473 |
| Sensitivity analysis 1 (Mantel-Haenszel method)^a^ | | | | | |  | 0.37 [0.13; 1.06]; 0.063 |
| Sensitivity analysis 2 (beta binomial model)^a^ | | | | | |  | 0.30 [0.07; 1.26]; 0.100 |
| a: IQWiG´s own calculation  CI: confidence interval; n.a.: not available; n: number of evaluated women with a rhesus-positive newborn; OR: odds ratio | | | | | | | |
